# Comparing practice characteristics and quality outcomes between parkrun and non-parkrun GP practices in England, with a focus on socioeconomic equity: a cross-sectional study

**DOI:** 10.1186/s12889-026-27560-5

**Published:** 2026-05-12

**Authors:** Callum Leese, Rosina Cross, Robert Mann, Emma Cockcroft, Jerome Mayaud

**Affiliations:** 1 Population Health and Genomics Department, University of Dundee, Dundee, UK; 2 Primary Care Research Group, College of Medicine and Health, University of Exeter, Exeter, UK; 3 Department of Public Health and Sport Sciences, University of Exeter, Exeter, UK; 4 Department of Health and Community Sciences Department, University of Exeter, Exeter, UK; 5 Third Variable Ltd, Aberfeldy, UK

**Keywords:** primary care, social prescribing, health equity, socioeconomic deprivation, quality improvement, physical activity, health promotion

## Abstract

**Background:**

Physical activity has wide-ranging benefits, and evidence supports the effectiveness of its promotion in primary care. The *parkrun practice* initiative is a voluntary social prescribing initiative encouraging General Practitioner (GP) practices to promote participation in free, weekly community-based physical activity events. Although potential benefits have been reported, the equity of the initiative’s uptake and impact remains unexplored.

The aim of this study was to compare characteristics and quality outcomes between *parkrun* and non-*parkrun* GP practices in England, with particular attention to socioeconomic equity.

**Methods:**

A cross-sectional observational study of English GP practices using routinely collected, publicly available data.

Data were analysed for 6,185 practices, of which 1,665 were parkrun practices. Outcomes included Quality and Outcomes Framework (QOF) points (financial incentive-based quality indicators related to the delivery of evidence-based care), patient satisfaction (national patient experience survey), and Care Quality Commission (CQC) ratings (regulatory practice quality ratings). Multivariable regression models were performed, adjusting for socioeconomic deprivation, list size, population demographics, and urban–rural classification. Moderation and deprivation-stratified analyses explored equity effects.

**Results:**

*parkrun practices* were more likely to be located in less socioeconomically deprived areas and serve populations with higher proportions of older and White patients; statistically significant differences were observed across all practice characteristic variables except urban/rural classification. Regarding practice quality outcomes, after adjustment, compared to non-*parkrun practices*, *parkrun practices* were associated with statistically significant higher QOF scores (mean difference +0.92 percentage points, p<0.001), higher patient satisfaction (+3.1 percentage points, p<0.001), and increased odds of a higher CQC rating (OR 1.32, p<0.001). Interaction analyses found no evidence that these associations differed systematically by deprivation, although stratified analyses suggested weaker or absent effects at the extremes of deprivation.

**Conclusions:**

Affiliation with the *parkrun practice* initiative is associated with modestly better practice quality outcomes, but uptake is socially patterned. Without targeted adaptation and support, the initiative may risk reinforcing existing inequalities.

**Supplementary Information:**

The online version contains supplementary material available at 10.1186/s12889-026-27560-5.

## Background

Regular physical activity (PA) can have significant physical, psychological, and social health benefits. For example, PA has been shown to improve symptoms of depression, anxiety, and distress, which are commonly encountered in primary care settings [1–3] Because of these benefits, PA is recognised by the World Health Organisation (WHO) as a key factor in both the prevention and management of long-term health conditions [4]. In the United Kingdom (UK), the Chief Medical Officers (CMO) provide guidance on the type and duration of PA to improve and maintain health. For adults, the UK Chief Medical Officers recommends at least 150 min of moderate-to-vigorous physical activity per week, including muscle strengthening activities at least two days per week [5]. Despite the well-established benefits of PA, adherence to these CMO guidelines is poor [6]. Specific to the UK general population, around one in three adult men and one in two adult women do not meet the PA guidelines [7]. These figures are typically lower for people with common (and multiple) long-term health conditions [8]. The burden of ‘low levels of PA’ (LPA) is a significant and growing global public health challenge. Large global analyses estimate that LPA is now responsible for around 0.8 million deaths and more than 15 million years of healthy life lost every year, and that, without action, almost 500 million new cases of preventable non-communicable diseases could occur between 2020 and 2030 due to inactivity [9, 10].

Increasing PA at the population level is a recognised public health priority, particularly among people with long-term conditions, who often have low levels of PA. One large-scale and community-based initiative that supports increases in population-level PA is *parkrun*. *parkrun* is a charity that delivers free, weekly, and timed five-kilometre walks or runs for all ages in parks and green spaces across the UK (and in 21 other countries around the world), with over 11 million registered participants worldwide [11]. These community events are organised and delivered by trained local volunteers and enabled through partnerships with national (e.g., health organisations) and local (e.g., councils).

In 2018, the Royal College of General Practitioners (RCGP) and parkrun launched the *parkrun practice* initiative to harness this community asset within primary care in the UK. The initiative encourages General Practitioner (GP) practices to link with their local *parkrun* event and to sign up as “p*arkrun practice*s”, with the intention that staff will promote *parkrun* to patients as a way to increase PA and social connection. The parkrun practice initiative is voluntary (no financial incentives) and deliberately flexible (allowing for tailored implementation). Implementation oversight is provided by the Royal College of General Practitioners (UK). Over 2,000 practices now promote *parkrun* to patients across the UK, and the scheme has been adopted internationally. Existing research into the potential benefits of the *parkrun practice* initiative indicates several potential benefits, including improved mental and physical health in patients and staff, and enhanced sense of community [12].

However, participation in the *parkrun practice* initiative is voluntary and depends on practice capacity, motivation, and local context. Practices in more socioeconomically deprived areas often experience greater workload pressures and resource constraints, which can limit their ability to engage with voluntary programmes such as the parkrun practice initiative [13]. This is particularly concerning given that people living in more socioeconomically deprived areas are the least likely to be physically active [7], increasing the risk that such initiatives may preferentially benefit more affluent populations and inadvertently widen health inequalities.

In England, primary care performance is evaluated using several established metrics, including patient satisfaction scores, the Quality and Outcomes Framework (QOF), and Care Quality Commission (CQC) ratings. Patient satisfaction scores are derived from standardized survey instruments capturing patients’ experiences of care. The QOF is a pay-for-performance scheme that assesses clinical quality across a range of disease areas and public health indicators, with achievement influencing general practice remuneration. The CQC provides independent regulatory oversight, conducting inspections and assigning ratings that reflect the overall quality and safety of care provision.

The aim of this study was, therefore, to explore the differences in characteristics and quality outcomes of *parkrun* practices versus non-*parkrun* practices in England, with a particular focus on health equity. The findings are intended to inform the future development and implementation of the *parkrun practice* initiative to ensure equitable reach, uptake, and benefit across diverse practice populations.

## Methods

### Study design, datasets and variables

The methodological approach is similar to that adopted in a previous study exploring the impact on quality outcomes of a different primary care-based initiative to promote PA [14]. We adopted a cross-sectional observational design, utilising routinely collected, publicly available datasets (via Public Health England) for GP practices in England. This approach has previously been used for examining population-level associations between physical activity related exposures and health system outcomes [15]. As of September 2025, there were 6,229 GP Practices in England [16], of which 2,137 were *parkrun practices*. Following data cleaning and the removal of any GP practices with incomplete data (*n* = 44), our final sample contained 6,185 practices, of which 1,665 (26.9%) were *parkrun practices* and 4,520 (73.1%) were non-*parkrun* practices.

The independent and outcome variables used in this study are listed in Table 1. We used the most recent publicly available datasets for each variable; while there were discrepancies between the time periods at which different variables were measured, we used the same time period consistently within each variable (except for the Care Quality Commission (CQC) ratings dataset, in which some practices had been rated in 2024 whereas others had been rated in 2015). All variables were specified a priori and drawn exclusively from routinely collected Public Health England Fingertips indicators, selected on the basis of theoretical relevance and prior literature rather than post-hoc exploration. In particular, measures of socioeconomic deprivation and rural–urban classification were included to capture complementary aspects of population context. These indicators are reported at the practice level using patient-level aggregation of Lower Layer Super Output Areas (LSOAs), as reported by the UK Office for National Statistics [17].

**Table 1:** Key characteristics of variables used in this study

Variable type	Variable	Data source	Data time period	Data type	Description
Independent	*parkrun practice* status	RCGP *parkrun* Practice List	Sep 2025	Binary (0 = No, 1 = Yes)	Whether or not a GP practice has *parkrun* accreditation.
Independent	IMD composite score	English Index of Multiple Deprivation (IMD)	2019	Numerical	An overall measure of socioeconomic deprivation at the LSOA level, combining 7 measures of different deprivation domains.
Independent	IMD decile	English Index of Multiple Deprivation (IMD)	2019	Ordinal (0–10)	Within-sample calculation of IMD decile based on raw IMD data.
Independent	Practice list size	Quality and Outcomes Framework (QOF)	2022–2023	Numerical	Number of people registered to the GP surgery. Accounts for structural differences between practices.
Independent	Practice geography	Rural Urban Classification (RUC)	2025	Categorical (Urban; Rural)	Standardised method for categorising LSOA geographies as either rural or urban, based on address density, physical settlement form, population size and relative access to major towns and cities.
Independent	Age group count	Practice Age and Gender Distribution	2024	Categorical (Children = 0–9 yrs; Adolescents = 10–19 yrs; Adults = 20–64 yrs; Older adults = 65 + yrs)	Count of patients at each practice, by age group (genders and ethnicity combined).
Independent	Sex count	Practice Age and Gender Distribution	2024	Categorical (Female; Male)	Count of patients at each practice, by sex (ages and ethnicity combined).
Independent	Ethnicity count	Practice Age and Gender Distribution	2024	Categorical (White; Asian / Asian British; Black; Mixed; Other).	Count of patients at each practice, by ethnicity (ages and gender combined).
Outcome	Patient satisfaction score	GP Patient Survey (GPPS)	2023	Numerical (%)	% who have a positive experience of their GP practice. Direct measure of patient experience and overall perception of care quality.
Outcome	Total QOF points	Quality and Outcomes Framework (QOF)	2022–2023	Numerical	Comprehensive indicator of clinical performance across various disease areas and public health.
Outcome	CQC rating	Care Quality Commission (CQC) Ratings	2015–2024	Ordinal (1 = Inadequate; 2 = Requires improvement; 3 = Good; 4 = Outstanding)	Independent regulatory assessment of safety, effectiveness, caring, responsiveness, and leadership.

To provide flexibility in our analysis, and to allow us to handle potential non-linear relationships, we:

- Calculated Index of Multiple Deprivation (IMD) deciles from our final study sample, by splitting the IMD scores into ten distinct categories, from ‘most deprived’ (decile 1) to ‘least deprived’ (decile 10).
- Amalgamated 5-year age group categories reported by the Office for Health Improvement and Disparities (OHID) into four age brackets, as recommended by Diaz et al. [18], to generate meaningful statistical analyses: [1] children (0–9 yrs); [2] adolescents (10–19 yrs); [3] adults (20–64 yrs); and [4] older adults (65 + yrs).
- Combined ethnicity classifications (*n* = 20) in the UK census into five categories, as recommended by the UK government [18], to simplify our analysis: [1] White; [2] Asian / Asian British; [3] Black; [4] Mixed; and [5] Other.

### Statistical analysis

#### Descriptive statistics

We summarised the characteristics of *parkrun practices* versus non-parkrun practices across all variables, using descriptive statistics appropriate to variable type, and assessed continuous variables for normality to guide subsequent analyses.

#### Bivariate analysis

Crude associations between *parkrun practice* and each quality outcome were explored using Mann-Whitney U tests for continuous variables, and Chi-squared tests for categorical variables.

#### Multivariable regression modelling

We used multivariable regression models to assess the independent association between parkrun practices versus non-parkrun practices and each quality outcome, adjusting for confounders. Ordinary Least Squares regression was applied to continuous outcomes (Quality and Outcome Framework (QOF) points and patient satisfaction), and ordinal logistic regression (OLR) to the ordinal outcome (CQC rating). A Brant test was performed to verify the proportional odds assumption for OLR. The test indicated that the strength of the association varied only slightly across thresholds (β = 0.74–0.89) and was consistently positive; we therefore consider the OLR as a parsimonious summary of the relationship. Ordinal model results are presented as odds ratios, representing the change in odds of achieving a higher CQC rating per one-unit increase in each predictor.

To address multicollinearity, we conducted iterative variance inflation factor (VIF) analysis, removing variables with pairwise correlations > 0.6 and retaining those with superior model fit until all VIFs were < 5. The final model included parkrun practice status, IMD score, list size, urban–rural classification, proportion of female patients, proportion of older adults and proportion of White ethnicity; other age and ethnicity variables were excluded.

Given that the three outcome variables represent conceptually distinct dimensions of practice quality, no correction for multiple comparisons was applied. We nonetheless note that all three primary associations would remain statistically significant under a Bonferroni-corrected threshold (α = 0.017).

#### Social equity analysis

To determine whether the effect of being a *parkrun practice* on quality outcomes significantly differs from non-parkrun practices across the spectrum of socioeconomic deprivation, we conducted moderation analysis by introducing a new variable to act as a moderator in our regression models: IMD score x *parkrun practice* status. Statistically significant interaction would indicate that the relationship between whether a practices is a *parkrun practice* or not and the outcome is not constant, and may be socially inequitable. To explore potential inequities even more deeply, we stratified our social equity analysis by testing the independent effect of *parkrun practice*s versus non-*parkrun practices* on each outcome variable within each IMD decile.

## Results

**Fig. 1:**
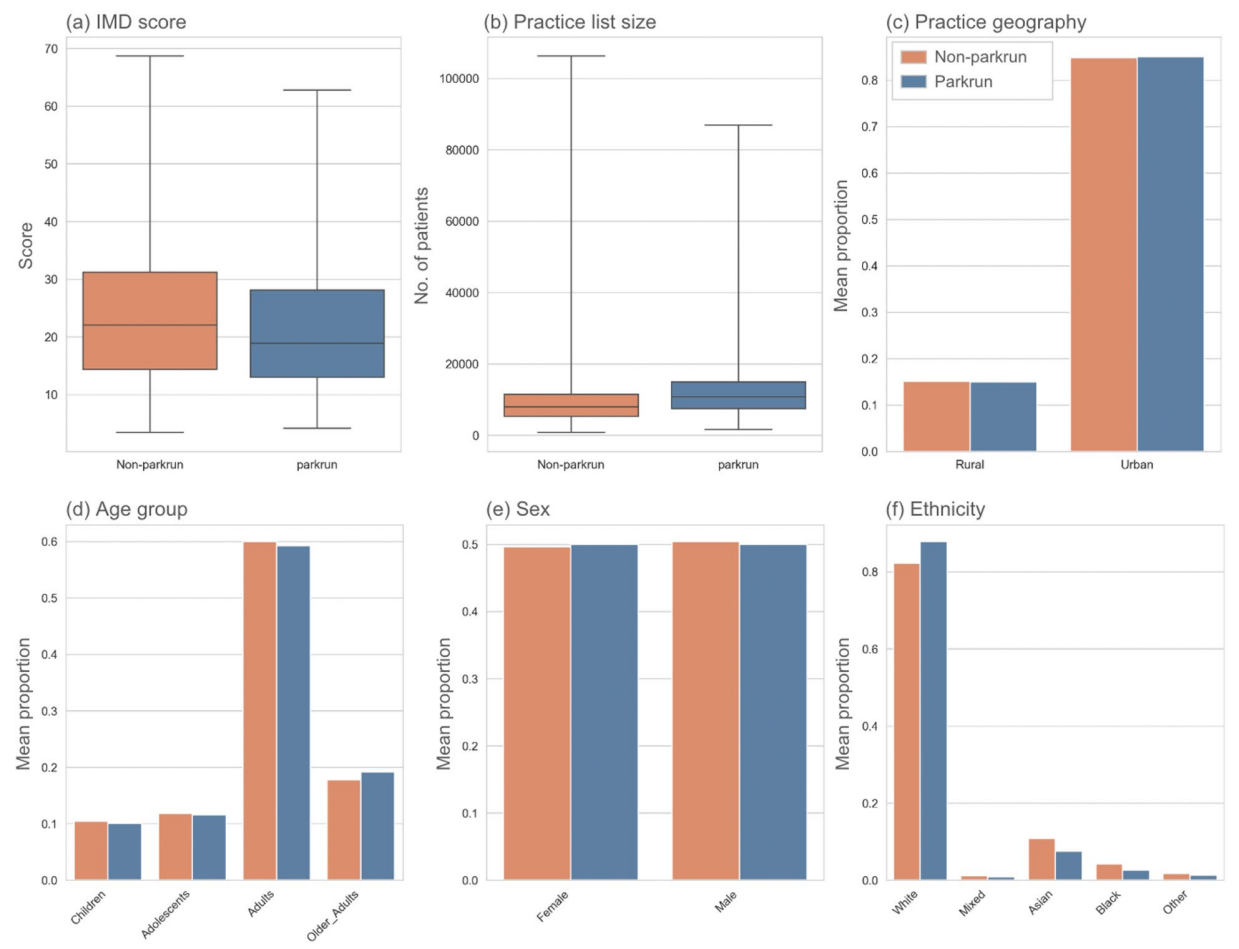
Distribution of independent variables, comparing parkrun (blue) and non-parkrun (orange) practices: **a** IMD score; **b** practice list size; **c** practice geography; **d** age group; **e** sex; and (**f**) ethnicity. Box-and-whisker plots in panels (**a**) and (**b**) display median, upper and lower quartile as box, and minimum and maximum values as whiskers

The distribution of the six independent variables chosen for this study are shown in Fig. 1. Result summaries from non-parametric Mann-Whitney U tests (to detect differences between *parkrun* and non-parkrun practices) are presented in Table 2.

**Table 2:** Mann-Whitney U test results for independent variables

Independent variable	parkrun median	Non-parkrun median	Median difference	*p* value
IMD score	18.9	22.1	-3.2	< 0.001
List size	10,781	7945	2836	< 0.001
Females proportion	0.505	0.501	0.004	< 0.001
Age: Children (proportion)	0.100	0.103	-0.003	< 0.001
Age: Adolescents (proportion)	0.115	0.116	-0.001	< 0.001
Age: Adults (proportion)	0.581	0.587	–0.006	< 0.001
Age: Older adults (proportion)	0.194	0.177	0.017	< 0.001
Ethnicity: White (proportion)	0.988	0.960	0.028	< 0.001
Ethnicity: Mixed (proportion)	0.0	0.004	-0.004	< 0.001
Ethnicity: Asian (proportion)	0.0	0.013	-0.013	< 0.001
Ethnicity: Black (proportion)	0.0	0.0	0.0	< 0.001
Ethnicity: Other (proportion)	0.0	0.0	0.0	< 0.001

*parkrun practices* had a statistically significantly lower IMD score (M_diff_ = -3.2, *p* < 0.001) than non-*parkrun practices* (i.e. *parkrun practices* were established in less socioeconomically deprived areas), and *parkrun practices* had a significantly larger list size (M_diff_ = 2826, *p* < 0.001). *parkrun practices* also had a slightly higher proportion of females (M_diff_ = 0.004, *p* < 0.001). A Chi-squared test showed that there was no significant difference in the urban-rural split of practices (χ^2^ = 0.024, *p* = 0.877). The differences in the proportion of all four age groups were statistically significant, with the largest difference being that *parkrun practices* have a higher proportion of older adults (M_diff_ = 0.017, *p* < 0.001). For ethnicity, *parkrun practices* had a higher proportion of White (M_diff_ = 0.028, *p* < 0.001) patients, and a lower proportion of Mixed (M_diff_ = -0.004, *p* < 0.001) and Asian (M_diff_ = -0.013, *p* < 0.001) patients.

### Quality outcomes

**Fig. 2:**
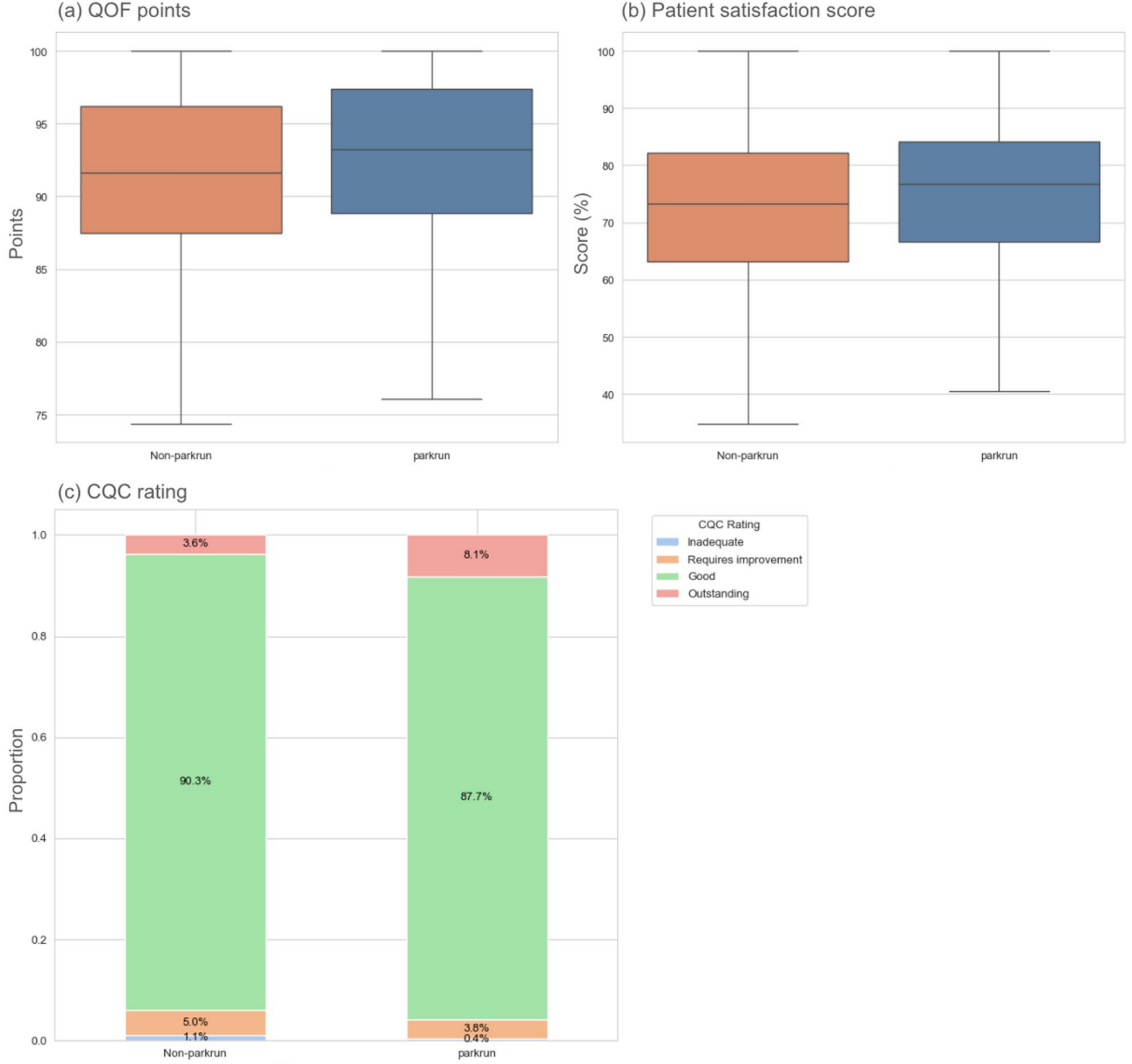
Distribution of outcome variables, comparing parkrun (blue) and non-parkrun (orange) practices: **a** QOF points; **b** patient satisfaction scores; **c** CQC rating. Box-and-whisker plots in panels (**a**) and (**b**) display median, upper and lower quartile as box, and minimum and maximum values as whiskers

Figure 2 displays the distribution of the three outcome variables used in this study. Shapiro-Wilk tests on the outcome variables indicated significant non-normality (*p* < 0.001 in all three cases). However, our large sample size (*n* = 6,185) provides sufficient robustness against the violation of the normality assumption for regressions and T-tests, based on the Central Limit Theorem.

T-tests reveal that *parkrun practices* had a statistically significantly higher QOF score than non-*parkrun practices* (*t* = 5.488, *p* < 0.001), as well as a significantly higher patient satisfaction score (*t* = 7.818, *p* < 0.001). Chi-squared testing showed that *parkrun practices* also had a significantly higher CQC rating than non-*parkrun practices* (χ^2^ = 60.929, *p* < 0.001), with a notably larger proportion of *parkrun practices* achieving an “Outstanding” rating (Fig. 2c).

### Multivariable regression modelling

Table 3 summarises the adjusted associations between parkrun practices and non-parkrun practices, practice characteristics, and each outcome.

**Table 3:** Results from main effects multivariable regression modelling (without moderation). Coefficients for OLS Models represent the change in the outcome variable for a one-unit increase in the predictor. Odds Ratios (OR) for the CQC Model represent the factor change in the odds of achieving a higher CQC rating. Significance: *** *p* < 0.001; ** *p* < 0.01; * *p* < 0.05

	QOF points(OLS)	Patient satisfaction score(OLS)	CQC rating(ordinal logit)
Statistic	Model fit		
Observations, N	6185	6185	6185
Adjusted R^2^	0.071	0.172	–
AIC	42,760	48,430	5229
Variable	β (95% CI)		Odds Ratio (95% CI)
*parkrun* status	0.916 (*p* < 0.001) *** (0.472, 1.359)	3.145 (*p* < 0.001) *** (2.448, 3.841)	1.302 (*p* < 0.001) *** (1.192, 1.423)
IMD score	-0.107 (*p* < 0.001) *** (-0.126, -0.087)	-0.152 (*p* < 0.001) *** (-0.182, -0.121)	0.999 (*p* = 0.484) (0.995, 1.002)
List size	0.000 (*p* < 0.001) *** (-0.001, 0.000)	-0.001 (*p* < 0.001) *** (-0.001, -0.001)	1.000 (*p* = 0.011) * (1.000, 1.000)
Urban	-0.095 (*p* = 0.763) (-0.710, 0.521)	-2.937 (*p* < 0.001) *** (-3.903, -1.971)	0.783 (*p* < 0.001) *** (0.693, 0.885)
Prop. female	23.664 (*p* < 0.001) *** (14.393, 32.936)	7.428 (*p* = 0.498) (-19.596, 9.526)	0.210 (*p* = 0.082) (0.036, 1.221)
Prop. older adults	12.528 (*p* < 0.001) *** (8.608, 16.448)	20.969 (*p* < 0.001) *** (14.812, 27.125)	1.787 (*p* = 0.144) (0.821, 3.890)
Prop. White	-0.774 (*p* = 0.138) (-1.796, 0.248)	7.711 (*p* < 0.001) *** (6.106, 9.316)	1.341 (*p* = 0.004) ** (1.096, 1.639)

### QOF points

OLS model explained a small proportion of variation (adjusted R^2^ = 0.071). After adjusting for practice and population characteristics, *parkrun practice* scored an average 0.92 additional QOF points compared with non-parkrun practices (*p* < 0.001). Practices serving more socioeconomically deprived populations had poorer QOF performance, with each one-point increase in IMD score associated with a 0.1-point reduction in QOF score (*p* < 0.001). Most other covariates were significant except urban classification and White proportion, with list size showing only a very small positive effect.

### Patient satisfaction

The patient satisfaction model explained a larger proportion of outcome variation (adjusted R² = 0.172). parkrun practices reported patient satisfaction rates that were, on average, 3.1% points higher than non-parkrun practices, even after adjustment for confounders (*p* < 0.001). Higher socioeconomic deprivation was associated with lower patient satisfaction, with a 0.2%-point decrease in satisfaction per IMD point (p *<* 0.001). Urban practices scored 2.9%-points lower than rural practices (*p* < 0.001). Practices with a higher proportion of older adults and those with a higher proportion of White ethnicity tended to have higher patient satisfaction scores (*p* < 0.001).

### CQC rating

In the logistic regression model, parkrun practice accreditation was associated with increased odds of achieving a higher CQC rating compared with non-parkrun practices (OR = 1.30, *p* < 0.001), after adjustment for covariates. IMD score was not significantly associated with CQC rating in the adjusted model. However, urban practices had decreased odds of achieving a superior rating compared with rural practices (OR = 0.78, *p* < 0.001), while practices with a higher proportion of White ethnicity had higher odds of a superior rating (OR = 1.34, *p* = 0.004).

### Social equity analysis

The moderation analysis is presented in Table 4. For all three models, the added interaction term (*parkrun* x IMD score) was not statistically significant, suggesting that the positive association between being a *parkrun* practice and outcome variables was uniform across all practices, regardless of how socioeconomically deprived their catchments were. Since the interaction term did not add explanatory power, we default to using the simpler (non-moderated) models as the basis for our primary findings.

**Table 4:** Results from multivariable regression modelling, with moderation. Significance: *** *p* < 0.001; ** *p* < 0.01; * *p* < 0.05

	QOF points(OLS)	Patient satisfaction score(OLS)	CQC rating(ordinal logit)
*Variable*	β (95% CI)		Odds Ratio (95% CI)
*parkrun* × IMD score	-0.026 (*p* = 0.187) (-0.065, 0.013)	-0.007 (*p* = 0.820) (-0.063, 0.054)	0.997 (*p* = 0.515) (0.990, 1.005)
*parkrun* status	1.477 (*p* = 0.003) ** (0.517, 2.438)	3.300 (*p* < 0.001) *** (1.791, 4.809)	1.378 (*p* = 0.001) *** (1.138, 1.667)
IMD score	-0.101 (*p* < 0.001) *** (-0.122, -0.079)	-0.150 (*p* < 0.001) *** (-0.184, -0.117)	0.999 (*p* = 0.725) (0.995, 1.004)
List size	0.000 (*p* < 0.001) *** (-0.001, 0.000)	-0.001 (*p* < 0.001) *** (-0.001, -0.000)	1.000 (*p* = 0.011) * (1.000, 1.000)
Urban	-0.101 (*p* = 0.747) (-0.717, 0.514)	-2.939 (*p* < 0.001) *** (-3.905, -1.973)	0.782 (*p* < 0.001) *** (0.692, 0.884)
Prop. female	22.636 (*p* < 0.001) *** (14.365, 32.907)	-5.043 (*p* = 0.497) (-19.605, 9.520)	0.211 (*p* = 0.083) (0.036, 1.222)
Prop. older adults	12.458 (*p* < 0.001) *** (8.537, 16.380)	20.949 (*p* < 0.001) *** (14.790, 27.108)	1.776 (*p* = 0.148) (0.816, 3.866)
Prop. White	-0.761 (*p* = 0.145) (-1.783, 0.262)	7.715 (*p* < 0.001) *** (6.109, 9.321)	1.343 (*p* = 0.004) ** (1.098, 1.642)

Table 5 presents results from the stratification analysis to test the independent effect of being a *parkrun* practice on each outcome (controlling for other covariates) within each IMD decile (decile 1 = “most deprived”, decile 10 = “least deprived”). The positive effects of being a *parkrun practice* on QOF achievement was statistically significant only in a less socioeconomically deprived segment of the population (decile 9), where *parkrun practices* were associated with QOF scores that were 1.7 points (*p* = 0.098) higher compared to non-*parkrun practices*.

**Table 5:** Results from decile stratification analysis. Significance: *** *p* < 0.001; ** *p* < 0.01; * *p* < 0.05. Coefficients represent the change in the outcome variable for a one-unit increase in the predictor. Odds Ratios for the CQC Model represent the factor change in the odds of achieving a higher CQC rating

Decile	QOF points (parkrun coefficient)	Patient satisfaction score (parkrun coefficient)	CQC rating (Odds ratio, 95% CI)
1 (most deprived)	-1.276 (*p* = 0.200)	0.921 (*p* = 0.512)	0.820 (*p* = 0.232)
2	1.639 (*p* = 0.075)	4.011 (*p* = 0.002) **	1.473 (*p* = 0.009) **
3	0.991 (*p* = 0.205)	4.045 (*p* < 0.001) ***	1.628 (*p* = 0.002) **
4	1.451 (*p* = 0.113)	3.192 (*p* = 0.013) *	1.206 (*p* = 0.225)
5	0.716 (*p* = 0.351)	3.042 (*p* = 0.006) **	1.232 (*p* = 0.139)
6	0.257 (*p* = 0.732)	3.408 (*p* = 0.003) **	1.606 (*p* = 0.002) **
7	0.659 (*p* = 0.371)	4.103 (*p* < 0.001) ***	1.313 (*p* = 0.049) **
8	1.139 (*p* = 0.098)	3.556 (*p* = 0.001) ***	1.610 (*p* < 0.001) ***
9	1.725 (*p* = 0.006) **	2.386 (*p* = 0.026) *	1.132 (*p* = 0.381)
10 (least deprived)	0.151 (*p* = 0.812)	1.449 (*p* = 0.157)	1.112 (*p* = 0.467)

The patient satisfaction results revealed a different stratification pattern, with statistically significant, positive relationships between being a *parkrun* practice and patient satisfaction spanning almost the entire spectrum of socioeconomic deprivation, except the extreme ends (deciles 1 and 10). The CQC results show that being a *parkrun* practice was associated with significantly increased odds of a higher CQC rating in the mid-range of socioeconomic deprivation (deciles 2, 3, 6, 7 and 8). Similar to patient satisfaction, being a *parkrun* practice had no significant effect at the extreme ends of the socioeconomic deprivation scale in this stratification analysis.

## Discussion

Findings from this national cross-sectional analysis indicate an association between *parkrun practice* affiliation and higher QOF points, patient satisfaction score and CQC rating. Compared with non-*parkrun practices*, *parkrun practices* had statistically significantly lower IMD scores, higher proportions of older adults and a higher proportion of white ethnicity. This implies evidence of socioeconomic inequities in the take-up of the *parkrun practice* initiative across English GP practices. However, our moderated regression models showed no evidence of inequity among practices that implemented the *parkrun practice* initiative. Subsequent decile stratification analysis suggested that *parkrun practice* accreditation may be linked to higher outcomes in the mid-range deciles (i.e. not at the extremes) but given the lack of interaction at the global level, these localised effects must be interpreted with caution.

Practices participating in the *parkrun practice* initiative tended to achieve higher outcomes across all three selected quality metrics compared with non-participating practices. The impact on QOF points, however, was relatively modest, with an average increase of only 0.93 points. Given that our QOF scores ranged from approximately 73% to 100%, this increase represents about 3.4% of the observed range. This relatively small effect may reflect an absence of current QOF indicators that explicitly reward the promotion of PA, with any observed association instead likely to reflect mediating factors. A plateau effect may also be present, whereby practices that are able to adopt the parkrun practice initiative already serve populations with relatively high baseline QOF scores, limiting the scope for further measurable improvement.

Patient satisfaction and CQC ratings, being more subjective measures, may better capture a practice’s commitment to holistic care, including support for PA. Our sensitivity analysis of CQC ratings before and after the introduction of the *parkrun practice* initiative suggests that the strong positive association observed in the main model was primarily driven by ratings awarded during the period of participation. This finding reduces the likelihood of reverse causality, whereby practices rated as ‘Outstanding’ are simply more likely to join the initiative, and instead indicates that the factors motivating practices to participate are concurrent with those contributing to recent regulatory success.

Our analysis operates at the practice rather than individual patient level, yet our finding that parkrun practices are more likely to be in less socioeconomically deprived areas aligns with documented inequalities in access to exercise interventions. Evidence from the Welsh National Exercise Referral Scheme offers a useful parallel [19]. Between 2008 and 2017, individuals living in the most socioeconomically deprived areas were not only less likely to be referred to the scheme, but -if referred- were also less likely to participate compared with those from less socioeconomically deprived areas. In contrast, an earlier publication from 2008 reported that general practices in more affluent areas were less likely to refer patients to exercise referral schemes (adjusted risk ratio for trend across socioeconomic deprivation quintiles 0.84; 95% confidence interval [CI] 0.76 to 0.93), suggesting that referral patterns and contextual influences may change over time and differ by initiative [20]. Collectively, these findings demonstrate that initiatives seeking to increase PA in populations at greatest need must attend not only to programme design, but also to the structural and contextual factors that influence both practice engagement and patient participation across socioeconomic gradients.

Although referral patterns for exercise interventions may evolve over time and differ by initiative [19, 20], cross-sectional studies consistently demonstrate that practices serving socioeconomically deprived populations have fewer resources and fewer GPs per patient- a long-recognised as the ‘inverse care law’ [13, 21]. These structural disadvantages contribute to lower patient satisfaction and poorer quality indicators [21]. Practices in socioeconomically deprived settings therefore face the dual challenge of higher demand and limited resources, making the equitable implementation of voluntary programmes particularly difficult [13].Our findings are consistent with this evidence, showing that *parkrun practices* are disproportionately clustered in more affluent areas, where population need is arguably lower.

While the *parkrun practice* initiative has been designed to be low-burden in an attempt to mitigate these barriers, previous research suggests that successful delivery nonetheless depends on staff time, capacity, and enthusiasm [22]. The absence of a statistically significant moderation effect in our analysis does not negate the presence of inequality; rather, the stratified results – showing weaker or absent effects at the extremes of socioeconomic deprivation – mirror patterns observed in other initiatives, such as the NHS Health Check programme [23], and underscore the importance of implementation context in shaping effectiveness.

Evidence from smoking cessation services and certain exercise referral schemes demonstrates that targeted approaches can improve equity in reach and uptake [20, 24]. The key challenge for the *parkrun practice* initiative is therefore to move beyond voluntary sign-up towards proactive, equity-focused implementation. For example, this could involve adopting a ‘proportionate universalism’ approach to implementation, with additional resources provided to those practices with the greatest need. Without targeted adaptation and additional support, the initiative risks reinforcing existing inequalities rather than reducing them.

Tailoring interventions for socioeconomically deprived populations needs to account for what is a multi-faceted problem, encompassing a large array of factors that can influence an individual’s ability to engage in regular PA. These may include individual factors (e.g. time, social support, self-efficacy, pre-existing health conditions) and systemic factors (e.g. presence of – and access to – safe places to engage in PA, affordability, cultural and social norms) [25, 26]. Further efforts need to be made to adapt and implement the *parkrun practice* initiative to account for as many of these factors as possible, aspiring for equity of distribution. Practical examples may include supporting social prescribers to implement and establish the initiative and/or working with local authorities to provide investment in transport links to events and additional financial support to ensure equitable and sustainable delivery.

Further work is also required to assess patient outcomes of the initiative across the socioeconomic deprivation spectrum. Equality of implementation of the initiative does not mean equality in outcomes, as demonstrated by the NHS diabetes prevention programme, which showed that those from minority ethnic backgrounds and lower socioeconomic backgrounds lost less weight and experienced lower glycated haemoglobin reduction [27]. However, research has shown that interventions promoting PA in primary care are effective across a diverse patient group, including those experiencing socioeconomic disadvantage [28].

### Strengths and limitations

A key strength of this study is its use of a large, nationally representative sample of English GP practices (*n* = 6,185) with routinely collected, publicly available data, enabling a robust examination of *parkrun practice* status across diverse practice characteristics and socioeconomic deprivation contexts. The study directly addresses an important equity gap by stratifying analyses across the full spectrum of socioeconomic deprivation. Furthermore, the inclusion of multiple quality outcome measures (QOF, patient satisfaction, CQC ratings) and adjustment for relevant confounders (socioeconomic deprivation, list size, demographics, urban–rural classification) provides a comprehensive assessment of associations and reduces the risk of confounding by observable variables.

We acknowledge several limitations. First, the observational design allows identification of associations but cannot establish causal relationships. Residual confounding by unmeasured variables such as practice motivation, staff expertise, or prior quality improvement engagement therefore remains. Second, the voluntary nature of parkrun practice introduces selection bias. Practices achieving accreditation may be inherently more motivated or well-resourced, meaning observed associations may partly reflect these characteristics rather than participation in the initiative itself. Third, our outcome measures (QOF, patient satisfaction, CQC ratings) are imperfect proxies that may not capture patient-level physical activity or health outcomes. We cannot assess whether practice affiliation translates into increased patient engagement with parkrun events. Fourth, temporal misalignment in datasets (some CQC assessments predating the initiative) may limit precision of comparisons. Finally, we operationalised parkrun status as binary without assessing implementation fidelity. Variation in quality of adoption may influence associations, particularly as practices in socioeconomically deprived areas facing greater workload pressure may have lower implementation quality despite accreditation.

## Conclusions

While these findings suggest that engagement with a community-based PA initiative may be linked to markers of improved practice performance (QOF score and patient satisfaction and CQC), the demographic profile of *parkrun practices* - larger list sizes, less socioeconomically deprived catchments and a higher proportion of older White patients - highlights important inequities in uptake. Given that physical inactivity and its associated long-term conditions are more prevalent in socioeconomically deprived and ethnically diverse populations, there is a possible risk that this initiative may widen existing health inequalities. The *parkrun practice* initiative may require targeted adaptation to ensure equitable uptake across socioeconomic and ethnic populations in order to avoid widening existing inequalities. Future research should aim to assess patient-level outcomes across the IMD gradient and implementation fidelity across socioeconomic deprivation gradients.

## Supplementary Information

Supplementary Material 1.

## Data Availability

Availability of data and materials: The data used in this study were obtained from publicly available datasets.Parkrun practice data: https://www.google.com/maps/d/u/0/viewer?mid=1nTMDcG5tS4rPKBqzEvAfHCBXFyq70uk&amp;ll=52.638330763068545%2C3.5621635673451424&amp;z=6GP Practice Profiles in England: https://fingertips.phe.org.uk/profile/general-practice/data#page/9/gid/2000005/pat/204/par/U89141/ati/7/are/A81001/iid/93468/age/28/sex/4/cat/-1/ctp/-1/yrr/1/cid/4/tbm/1Specific features: IMD Composite score: https://fingertips.phe.org.uk/profile/general-practice/data#page/9/gid/2000005/pat/204/par/U00000/ati/7/are/D82060/iid/93553/age/1/sex/4/cat/-1/ctp/-1/yrr/1/cid/4/tbm/1Patient satisfaction score (indicator id 93438): https://fingertips.phe.org.uk/profile/general-practice/data#page/9/gid/2000005/pat/204/par/U00000/ati/7/are/D82060/iid/93438/age/164/sex/4/cat/-1/ctp/-1/yrr/1/cid/4/tbm/1 List size (indicator id 114; it is only extractable via the API at the moment): https://fingertips.phe.org.uk/api/all_data/csv/by_indicator_id?indicator_ids=114&amp;child_area_type_id=7 % QOF points achieved (indicator id 295):https://fingertips.phe.org.uk/profile/general-practice/data#page/9/gid/2000005/pat/204/par/U00000/ati/7/are/D82060/iid/295/age/-1/sex/-1/cat/-1/ctp/-1/yrr/1/cid/4/tbm/1.

## Abbreviations

PA: Physical Activity
GP: General Practitioner
QOF: Quality and Outcomes Framework
CQC: Care Quality Commission
OR: Odds Ratio
WHO: World Health Organisation
CMO: Chief Medical Officers
LPA: Low Levels of Physical Activity
RCGP: Royal College of General Practitioners
UK: United Kingdom
IMD: Index of Multiple Deprivation
OHID: Office for Health Improvement and Disparities
ORL: Ordinal Logistic Regression
VIF: Variance Inflation Factor
OLS: Ordinary Least Squares
