# Supplementary material for: Comparing practice characteristics and quality outcomes between parkrun and non-parkrun GP practices in England, with a focus on socioeconomic equity: a cross-sectional study
Source: BMC Public Health. 2026 May 12;26:2041. doi: 10.1186/s12889-026-27560-5 (PMC13334893; doi:10.1186/s12889-026-27560-5)
Supplement: Supplementary file 1 — Supplementary Material 1. [file 12889_2026_27560_MOESM1_ESM.docx]

**Consensus Reporting Items for Studies in Primary Care CRISP Checklist**

The CRISP Checklist^1^ can help researchers meet readers’ needs by including content that the primary care community feels is important for the validity, quality, and usefulness of primary care research reports.

The Checklist summarizes recommendations for authors to consider in crafting their report. Nothing is intended to limit the effective or creative reporting of research. Authors and editors make final decisions.

Primary care research involves a wide variety of methods, study designs, topics, and settings. The Checklist covers this broad spectrum and therefore, not all items apply to all studies. Some items may not apply to your study. Some may ask for information that is not available. Check such items off and use the rest of the Checklist in whatever way helps you improve *your* report of *your* research.

How to use the CRISP Checklist:

- Each item is listed. Please respond to each item, even if it is not included in your report.
- Check if the item is included in your report: yes, no, or not applicable to your study.
- If the item applies to the study design but is not included in the report, please provide a brief explanation in the “Notes” section.
- The “Suggested Section” indicates where the item usually appears in a research report following the IMRaD format (Introduction, Methods, Results, and Discussion). These are suggestions only. If the item is in a different section of your report, you might add that in “Notes.”
- You can note the location of the item in your report by line, page, or section in “Notes.”

For more information plus an explanation and examples of each item, please see the supplemental guidance in the Appendix to the CRISP Statement.^2^

You may choose to submit the completed Checklist along with your manuscript to help editors and reviewers see how you have included the suggested items in your research report. Authors should also consider using other reporting guidelines that are appropriate for their study and report. (See Table 2.^1^) Some CRISP items may overlap with other guidelines.

For more information, see **CRISP:** <https://crisp-pc.org/>

**References:**

1. Improving the Reporting of Primary Care Research: Consensus Reporting Items for Studies in Primary Care—the CRISP Statement. William R. Phillips, Elizabeth Sturgiss, Paul Glasziou, Tim C. olde Hartman, Aaron M. Orkin, Pallavi Prathivadi, Joanne Reeve, Grant M. Russell, and Chris van Weel. *Annals of Family Medicine* November 2023, 21 (6) 549-555; DOI: <https://doi.org/10.1370/afm.3029>
2. Supplemental Appendix. Consensus Reporting Items for Studies in Primary Care (CRISP) Explanation and Examples Guide. <https://www.annfammed.org/content/annalsfm/suppl/2023/10/02/afm.3029.DC1/Phillips-Supp-App-Table-2023.pdf>

| **Reporting Item - 1** | **Included?** | | | **Section*** | | **Notes** |  |
| --- | --- | --- | --- | --- | --- | --- | --- |
|  | **Y** | **N** | **N/A** |  |  | | |
| **1. Include “primary care” and/or discipline-specific terms in the title, abstract, and/or key words.** | **Y** |  |  | I | **Title** | | |

| **Reporting Item - 2** | **Included?** | | | **Section*** | **Notes** |
| --- | --- | --- | --- | --- | --- |
|  | **Y** | **N** | **N/A** |  |  |
| **2. Describe the study rationale and importance for primary care.** | | | | | |
| **2a.** Explain the rationale for the research question and how it relates to primary care. | **Y** |  |  | I | Pg 6 |
| **2b.** Describe the importance or relevance of the topic under study in the primary care setting. | **Y** |  |  | I | Pg 5 |
| **2c.** Identify any theory, model, or framework used, and explain why it is appropriate to the research question in primary care. | **Y** |  |  | I | Pg 6 and 7 |

| **Reporting Item - 3** | **Included?** | | | **Section*** | **Notes** |
| --- | --- | --- | --- | --- | --- |
|  | **Y** | **N** | **N/A** |  |  |
| **3. Describe the research team’s primary care experience and collaboration.** | | | | | |
| **3a.** Describe the research team’s expertise and experience in primary care practice and/or research. | **Y** |  |  | M | **References 14 and 22 highlight teams expertise in area** |
| **3b.** Describe whether and how primary care patients, practicing clinicians, community members, or other stakeholders were involved in the research process. |  |  | **N/A** | M |  |

| **Reporting Item - 4** | **Included?** | | | **Section*** | **Notes** |
| --- | --- | --- | --- | --- | --- |
|  | **Y** | **N** | **N/A** |  |  |
| **4. Describe the study participants and populations in the context of primary care.** | | | | | |
| **4a.** Use person-focused language to refer to the research populations and participants, or use terms based on patient preferences |  |  | **N/A** | R | **Refer to parkrun practices versus non-parkrun practices** |
| **4b.** If reporting personal characteristics of participants, report the source of the data, the rationale for using it, and the rationale for any classifications used. |  |  | **N/A** | R |  |
| **4c.** Describe the participants and populations in sufficient detail to allow comparison to other primary care patient populations. | **Y** |  |  | R |  |
| **4d.** Specify whether participants have pre-existing therapeutic relationships with the clinical team or are new patients. |  |  | **N/A** | M/R |  |

| **Reporting Item - 5** | **Included?** | | | **Section*** | **Notes** |
| --- | --- | --- | --- | --- | --- |
|  | **Y** | **N** | **N/A** |  |  |
| **5. Describe the conditions under study in the context of primary care.** | | | | | |
| **5a.** Describe whether the condition under study is acute or chronic. |  |  | **N/A** | M/R |  |
| **5b.** Report how multimorbidity is considered and how it might affect interpretation of the study findings/ results. |  |  | **N/A** | M |  |

| **Reporting Item - 6** | **Included?** | | | **Section*** | **Notes** |
| --- | --- | --- | --- | --- | --- |
|  | **Y** | **N** | **N/A** |  |  |
| **6. Describe the clinical encounter under study in the context of primary care.** | | | | | |
| **6a.** Specify whether the study focus is an isolated clinical encounter or a longitudinal course of care. If it is an isolated clinical encounter, specify whether it is the first visit or a follow-up visit for the condition under study | **Y** |  |  | M | **Pg 6 and 7** |

| **Reporting Item - 7** | **Included?** | | | **Section*** | **Notes** |
| --- | --- | --- | --- | --- | --- |
|  | **Y** | **N** | **N/A** |  |  |
| **7. Describe the patient care team.** | | | | | |
| **7a.** If care is delivered by teams, describe the team members and their roles. |  |  | **N/A** | R |  |
| **7b.** For each clinician category, report profession, specialty, and qualifications. |  |  | **N/A** | R |  |

| **Reporting Item - 8** | **Included?** | | | **Section*** | **Notes** |
| --- | --- | --- | --- | --- | --- |
|  | **Y** | **N** | **N/A** |  |  |
| **8. Describe the study interventions in the context of primary care.** | | | | | |
| **8a.** Describe interventions and their implementation in sufficient detail to enable the reader to assess applicability in their own setting. | **Y** |  |  | M | Pg 4 and 5 |
| **8b.** Describe any clustering or grouping of patients, participants, clinicians, teams, or practices, and how it was addressed in the analysis. | **Y** |  |  | M/R | Pg 6 and 7 |
| **8c.** Describe the health care system in sufficient detail to allow comparisons to other systems. | **Y** |  |  | I/D | Pg 5 |

| **Reporting Item - 9** | **Included?** | | | **Section*** | **Notes** |
| --- | --- | --- | --- | --- | --- |
|  | **Y** | **N** | **N/A** |  |  |
| **9. Describe study measures used and their relevance to primary care.** | | | | | |
| **9a.** Report whether study measurement tools have been validated in primary care populations or settings. | **Y** |  |  | M | **Pg 6 and 7** |
| **9b.** Describe how the measurement tools used are meaningful to primary care patients and their care. | **Y** |  |  | M | **Pg 6 and 7. Also reference in discussion** |
| **9c.** Report findings/results to be clinically interpretable by primary care clinicians and patients. | **Y** |  |  | R |  |

| **Reporting Item - 10** | **Included?** | | | **Section*** | **Notes** |
| --- | --- | --- | --- | --- | --- |
|  | **Y** | **N** | **N/A** |  |  |
| **10. Discuss the meaning of study findings/results in the context of primary care.** | | | | | |
| **10a.** Discuss implications of the study findings/results for research, patient care, education, and policy with specific focus on primary care. | **Y** |  |  | D |  |
| **10b.** Discuss the implications of study recommendations on demands and priorities in primary care practice. | **Y** |  |  | D |  |
| **10c.** Comment on any research processes that might influence the applicability of the study findings/results in diverse primary care settings. | **Y** |  |  | D |  |

***Section:** I = Introduction, M = Method, R = Results, D = Discussion
